# Re: Artificial urinary sphincter for urinary incontinence after radical prostatectomy: a historical cohort from 2004 to 2015

**DOI:** 10.1590/S1677-5538.IBJU.2017.0074

**Published:** 2017

**Authors:** José Carlos Truzzi, Carlos Alberto Ricetto Sacomani, José Antônio Prezzoti

**Affiliations:** 1 Departamento de Uroneurologia, Sociedade Brasileira de Urologia, Rio de Janeiro, RJ, Brasil

*To the editor,*

The article entitled “Artificial urinary sphincter for urinary incontinence after radical prostatectomy: a historical cohort from 2004 to 2015” (1), presents results which interpretation deserves some remarks. This retrospective study was performed at Belo Horizonte (Minas Gerais, Brazil) involving a 11 year-period (2004-2015) in 15 different hospitals, being the procedure performed by 28 different surgeons. A simple mathematic analysis shows that there were less than eight procedures per year (7, 8/year), and that each doctor implanted a median of three sphincters in that period, corresponding in absolute numbers to 0.27 implants per year per physician. This value does not allow a correct learning curve for a complex procedure that demands technical expertise, wide knowledge of voiding dysfunctions and physiology of male urinary incontinence. Also, previous clinical data of included implanted patients were not presented. Parameters such as severity of post-prostatectomy incontinence, presence (or not) or detrusor hyperactivity, impaired bladder complacency, detrusor hypocontractility, urethral stenosis or stenosis of urethro-vesical anastomosis, ureteral reflux, diabetes mellitus, were not provided. When all patients are considered as a whole without consideration of the above mentioned parameters, there is a risk of interpretation of results of a heterogenous group of patients. Analysis was based in an administrative data bank of a health insurance company. There was no collection of data from the patient’s charts. Questions related to indication, evolution and results regarding the used technique were not available.

In relation to complications, it is possible to question if those mentioned as due to sphincter implantation were really related, among which urethral stenosis and spermatic cord torsion. Urethral stenosis and stenosis of urethro-vesical anastomosis may occur due to radical prostatectomy and not necessarily due to the sphincter implant. The mentioned testicular torsion (spermatic cord torsion) is not a complication of artificial sphincter implant. The candidate to artificial sphincter must be evaluated thoroughly before the surgical act. It is essential to understand the basic mechanisms of the functioning of artificial sphincter in order to operate it correctly.

Also, among the listed complications, even if graded as Clavien-Dindo III, transitory acute urinary retention usually occurs due to urethral edema related to urethral manipulation during surgery with high resolution success with bladder catheterization in less than 48 hours.

According to presented data, four (16.7%) of artificial sphincters showed mechanical problems. However, the authors stated that eight patients (33.3%) needed change of cuff due to malfunction and three (12.5%) had to change the regulator balloon. All these are usually caused by mechanical failure. There is an incompatibility of presented data: were there four or eleven failed sphincters?

When such complications are addressed altogether, without correct evaluation of patients (using only data obtained from charts) it is possible to criticize negatively an internationally accepted procedure, considered as gold-standard in the treatment of male urinary incontinence, used for more than 30 years and more that 100.000 documented implants. It is important to stress that artificial sphincter implant does not guarantee full continence recovery, but it is a treatment option for severe urinary incontinence, that improves quality of life of patients with involuntary urinary leakage. In that study, it was reported that 15.3% of patients remained incontinent following the sphincter implant. However, it is difficult to interpret this data, since there was no description of pre-operatory status, or if there was reduction of incontinence episodes or lowering of number of used pads following sphincter implant.

A recent review included 1.082 patients submitted to implantation of AMS-800 artificial sphincter, 78% following radical prostatectomy and 27% after pelvic radiation, with a median follow-up of more than five years. Primary implanted sphincter survival was 74% after 5 years and 41% at 15 years of follow-up (2). Other studies showed similar survival rates in shorter follow-ups periods, and more modest late survival rates (3, 4). But when it was applied the subjective satisfaction index, 73% of patients were satisfied or very satisfied with their continence (5). In a systematic review of literature concerning surgical treatment of post-radical prostatectomy urinary incontinence, conducted recently by Crivellaro et al., artificial sphincter presented the better positive results, but with wide variability margin (20% to 89%), followed by slings and urethral compressor model pro-ACT (3). In that same systematic review, urethral erosion was the most frequent complication of artificial sphincter implant, with an incidence of 3% to 7.4%; general infection rate after implant was up to 10% (3).

Previous exposure to radiation is classically considered a risk factor for complications, mainly erosion and local infection (6). However, Rivera et al. did not show any statistical difference in the number of removals of artificial sphincters, survival and rate of infection or erosion among 323 radiated patients from a total of 872 submitted to AMS-800 implant (7).

The authors stated that there are no Brazilian studies on the subject of artificial sphincters and they are wrong: Trigo Rocha et al. published at Urology journal (8) the results of 40 patients submitted to sphincter implant following post-radical prostatectomy incontinence followed-up to a medium of 53 months.

In conclusion, the article presented results contrary to the majority of national and international studies on the theme, and raises doubts on probable causes, such as local problems or the analyzed patient’s sample. It is interesting that the authors stablish which factors caused such unsatisfactory results. Also, it should have been stated in the “Conflict of Interests” (although mentioned in the authors identification) that the authors are members of a technical group associated to a private health institution.
